# Reactive Oxygen Species‐Induced Damage in Cryopreserved Bovine Sperm: Mitigation Strategies

**DOI:** 10.1002/vms3.71055

**Published:** 2026-06-29

**Authors:** Mst. Mahomudha Akhtar, Sakib Hossain, Afia Mahmuda Meem, Afiya Fairuz Lubaba, Md. Kamrul Hasan Kazal

**Affiliations:** ^1^ Department of Animal Breeding and Genetics Bangladesh Agricultural University Mymensingh Bangladesh; ^2^ Department of Biochemistry and Molecular Biology Bangladesh Agricultural University Mymensingh Bangladesh

**Keywords:** antioxidants, bovine sperm, cryopreservation, fertility, reactive oxygen species

## Abstract

**Background:**

The importance of sperm cryopreservation in cattle production is that it enables the flow of genetic variation and the speedy distribution of genetically superior bulls. Nevertheless, there are still considerable technological gaps because the quality of post‐thaw sperm is always poor and differs significantly across breeding bulls.

**Objectives:**

The aim of this review was to investigate the origin of reactive oxygen species (ROS) generation in the process of bovine sperm cryopreservation, discuss the effects of ROS‐induced injury, and present the current knowledge of the measures the researcher can take to alleviate the impact of oxidative stress.

**Methods:**

A thorough literature review was made by searching the relevant scientific databases with the keywords: bull sperm cryopreservation, ROS, oxidative stress, antioxidants, non‐antioxidants approaches and post‐thaw sperm quality.

**Results:**

Freeze–thaw stress causes extreme cellular and molecular stress and ROS overproduction, which is one of the major causes of sperm damage. The main causes of ROS production are changes in osmotic pressure, the presence of ice crystals, mitochondrial dysfunction and oxidative burst. In turn, excessive production of ROS causes lipid peroxidation of the plasma membrane, oxidation of proteins, fragmentation of the DNA and dysfunction of the mitochondria, which eventually decreases the sperm motility, viability and fertilization capacity. Enzymatic and non‐enzymatic antioxidants, plant extract, nanoparticles, mitochondria‐targeted antioxidants and non‐antioxidants approaches have proven to be effective in curbing oxidative damage.

**Conclusion:**

Prevention of oxidative imbalance in cryopreservation is an important factor that can increase the quality of post‐thaw sperm and increase the success of artificial insemination and in vitro fertilization programmes in cattle.

## Graphical abstract

Cryopreservation of bovine sperm results in overproduction of ROS due to osmotic pressure variations, ice crystals and dysfunction of mitochondria, which causes lipid peroxidation, fragmentation of DNA, protein damage and ultimately decline of post‐thaw sperm quality. Enzymatic and non‐enzymatic antioxidants, plant extract, mitochondria‐targeted antioxidants, nanoparticles and non‐antioxidant approaches are effective to counter the damages, thereby increasing the post‐thaw sperm quality and success rates of artificial insemination and in vitro fertility in cattle.

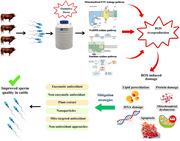

## 1 Introduction

Reactive oxygen species (ROS) are extremely reactive molecules produced from oxygen that can be either free radicals (present unpaired electrons) or non‐radicals. ROS include superoxide anions (O_2_•^−^), hydroxyl radical (HO•), nitric oxide (NO•) and peroxyl radical (ROO•) (Radi 2018), which have unpaired electrons. Other ROS with oxidizing characteristics, but not free radicals, include hydrogen peroxide (H_2_O_2_), peroxynitrite (ONOO^−^), singlet oxygen (^1^O_2_), and hypochlorous acid (HOCl) (Phaniendra et al. 2015). They are produced as a result of cellular metabolism (Sies et al. 2022), predominantly in the mitochondria and at physiological level play necessary roles in sperm function, for example, capacitation, hyperactivation and the acrosome reaction (Sapanidou et al. 2022). Nevertheless, elevated ROS production can overwhelm the intrinsic antioxidant defence systems, leading to oxidative stress that disrupts plasma membrane integrity, DNA firmness, mitochondrial efficiency, and ultimately loss of fertilizing potential (Dutta et al. 2019).

Bovine sperm are particularly vulnerable to ROS‐induced damage during cryopreservation because of their high content of polyunsaturated fatty acids (PUFAs) in the plasma membrane (Kujoana et al. 2024), which are highly susceptible to lipid peroxidation (LPO) (Wang et al. 2025). In addition, spermatozoa possess very limited cytoplasmic volume, resulting in a poor supply of intrinsic antioxidant enzymes such as superoxide dismutase (SOD), catalase (CAT), and glutathione peroxidase (GPx) (Fleming and Thomson 2025). During the freeze–thaw process, rapid changes in temperature, osmotic stress and ice crystal formation disrupt mitochondrial function, leading to excessive production of ROS, which is frequently deteriorating post‐thaw sperm quality (Sharafi et al. 2022).

To address these challenges, several strategies have been explored to mitigate oxidative stress and improve cryo‐survival. Antioxidants (enzymatic and non‐enzymatic) serve as the main defence mechanism against ROS by scavenging and neutralizing them (Tariq et al. 2015). In addition, medicinal plants and phytochemicals have gained attention for their natural antioxidant potential (Halil et al. 2024). More recently, nanoparticles have emerged as innovative approaches, offering targeted antioxidant delivery, enhanced ROS scavenging, and improved post‐thaw sperm survival (Hozyen et al. 2019; Alemdar and Tırpan 2023). Parallel to these methods, mitochondrial‐targeted antioxidants have demonstrated promising results in reducing oxidative stress and improving sperm cryo‐survival (Carneiro et al. 2018; Elkhawagah et al. 2024), and non‐antioxidant approaches, such as cryoprotectant optimization, oxygen tension control, removal of defective sperm, seminal plasma management and osmotic stress minimization, have also shown significant potential in enhancing sperm quality parameters. Here, the current review is aimed at the sources and mechanisms of ROS generation during bovine sperm cryopreservation and their effects on sperm quality and function. It also incorporates major categories of protective strategies—enzymatic and non‐enzymatic antioxidants, plant‐based extracts, nanoparticles, mitochondria‐targeted molecules and non‐antioxidants methods—into a critical framework. This review is the first synthesis to address the specific bovine systems in detail and up‐to‐date, unlike the previous reviews, which have reviewed the antioxidant supplementation and non‐antioxidants methods in sperm cryopreservation on a general or multi‐species scale separately (Sapanidou et al. 2023; Qamar et al. 2022).

## 2 ROS and Sperm Physiology

Good quality sperm is crucial for successful animal reproduction, but production of ROS is a major obstacle in this process (Zhang et al. 2025). ROS have both regulatory and detrimental effects on sperm physiology. Optimum ROS levels maintain a functional redox state and play crucial physiological roles in reproductive events such as sperm maturation, hyperactivation, acrosome reaction, and sperm‐oocyte fusion due to their short half‐life and limited diffusion (Upadhyay et al. 2022). The main functions of ROS in sperm physiology have been generally preserved across mammals, but bovine spermatozoa have species‐specific characteristics making them more susceptible to oxidative stress. In particular, the percentage of PUFAs in the membranes of bull sperm is extremely great, and the low size of cytoplasmic volume reduces the accessibility of endogenous antioxidant enzymes (Kujoana et al. 2024). Consequently, bovine sperm becomes more prone to LPO occasioned by the ROS, specifically during the cryopreservation procedures, which is why oxidative stress is one of the major issues in bovine reproductive technologies. Results of non‐bovine species, including human or mouse models, are cited in this study primarily either to indicate conserved molecular pathways or provide mechanistic context in areas where bovine‐specific data is limited. This evidence between species is not provided as necessarily applicable to bovine systems but as a starting point to make species‐specific conclusions. ROS are sub‐products of respiration derived mainly from mitochondrial Complexes I and III and from sperm activity (Giaretta et al. 2022; Zhang et al. 2024). Functionally competent spermatozoa generate higher levels of ROS, but excessive ROS or imbalance between ROS production and the antioxidant buffering capacity of seminal plasma can cause oxidative stress, which may irreversibly damage spermatozoa (Qamar et al. 2022). Oxidative stress due to excessive ROS production is known to hinder normal sperm function and influence infertility. ROS are also associated with damage to cellular lipids, proteins and sperm DNA, which can have profound implications for normal embryonic development and long‐term progeny health (Sies 2017).

## 3 Sources and Mechanisms of ROS Generation During Bovine Sperm Cryopreservation

**FIGURE 1:**
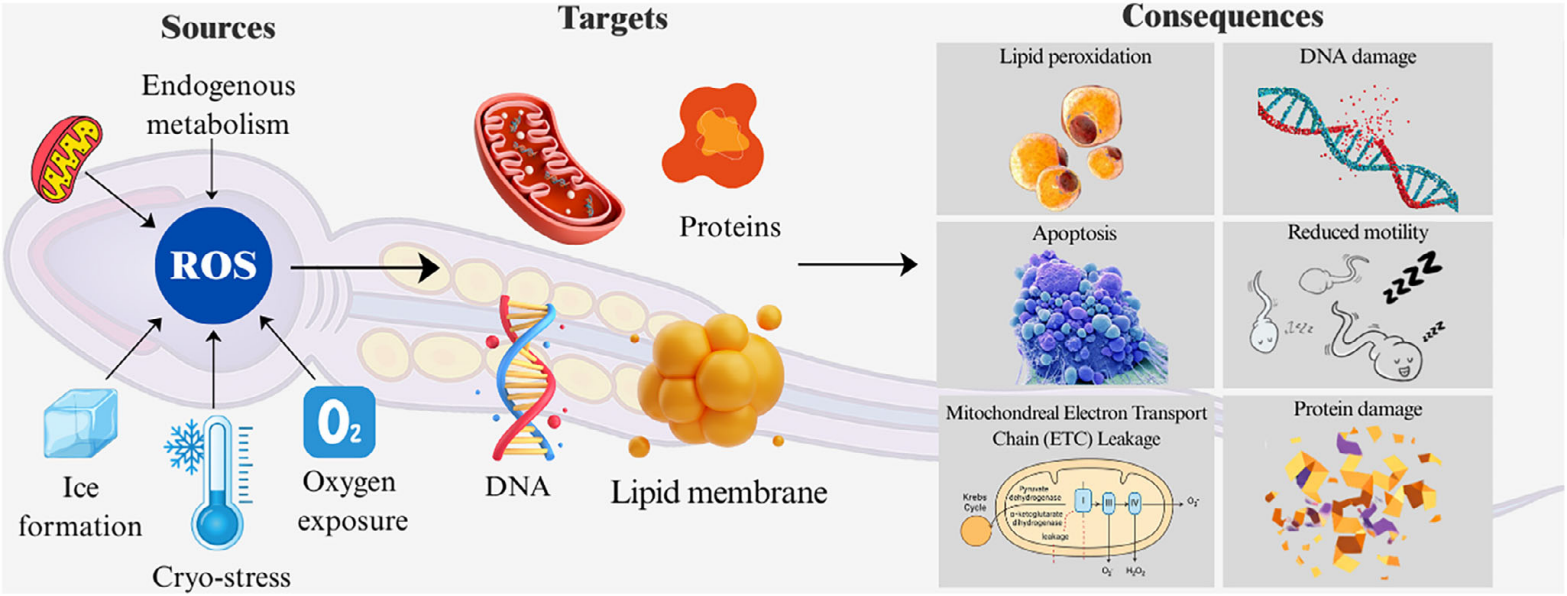
Mechanism of ROS‐induced damage in cryopreserved sperm. The figure depicts the major sources of ROS generation during the freeze–thaw process. These include electron leakage from the mitochondrial ETC, activation of NADPH oxidase (NOX), xanthine oxidase activity within the extender and oxidative bursts originating from leukocytes. Together, these pathways lead to an accumulation of intracellular ROS that exceeds the neutralizing capacity of endogenous antioxidant systems such as SOD, CAT, GPx and GSH. This imbalance initiates a series of oxidative injuries, including lipid peroxidation of the plasma membrane—resulting in decreased membrane fluidity and compromised acrosomal integrity—protein carbonylation that disrupts flagellar movement and metabolic activity, and DNA damage characterized by strand breaks and base modifications. Additionally, mitochondrial membrane potential is diminished, leading to reduced ATP production and the activation of apoptotic pathways. Ultimately, these alterations impair post‐thaw sperm quality and diminish fertilization capability. ROS, reactive oxygen species.

During cryopreservation, bovine spermatozoa undergo drastic physical and biochemical stress that promotes excessive generation of ROS. Several major sources and mechanisms are involved (Figure 1).

### 3.1 Mitochondrial Electron Transport Chain (ETC) Leakage

Both the Krebs cycle and the ETC of mammalian mitochondria have been identified to have a number of potential locations for the synthesis of superoxide and H_2_O_2_ (Tabassum et al. 2020). Under normal physiological conditions, approximately 0.2%–2% of the electrons in the ETC deviate from their usual transfer pathway and prematurely react with oxygen, resulting in the formation of superoxide or hydrogen peroxide (Zhao et al. 2019). Therefore, electron leakage describes the process in which electrons escape from the ETC before reaching Complex IV, where they would normally be reduced to water (Kasumov et al. 2015).

### 3.2 Nicotinamide Adenine Dinucleotide Phosphate (NADPH) Oxidase (NOX) System

Known to be obligate components of redox signalling, the NADPH oxidase (NOX) isoforms are a category of enzymes that generate O_2_•^−^ and H_2_O_2_ in various cell types (O'Flaherty 2025). It has been unravelled that NADPH oxidase 5 (NOX5) is the key source of ROS production in some mammalian spermatozoa, such as human and equine (Miguel‐Jiménez et al. 2021). Cold shock and calcium influx during freezing–thawing can stimulate this enzyme. It is necessary to add that there are few positive signs of the activity of NOX5 in bovine spermatozoa. The bovine NOX5 ortholog and its regulatory processes in cryopreservation can be different in relation to the described processes in human and equine sperms, and thus, species‐specific research is necessary.

### 3.3 Xanthine Oxidase (XO) Activity in Seminal Plasma/Extender

XO plays a key role in purine metabolism by converting purine nucleotides into uric acid. During cooling or freezing, reduced ATP levels activate XO in seminal plasma or extenders, which metabolizes hypoxanthine and xanthine into ROS, primarily superoxide (O_2_•^−^) and H_2_O_2_ (Aziz and Jamil 2023). Its function is to catalyse the terminal degradation of purine bases that generate uric acid as the last product of purine catabolism (Kimoloi 2018).

### 3.4 Dead or Damaged Spermatozoa

During the freezing–thawing process, cryopreservation inevitably leads to a proportion of spermatozoa being irreversibly damaged or killed. Dead spermatozoa have been suggested to represent the major ROS producers through an aromatic l‐amino acid oxidase (LAAO) pathway in semen. When sperm membrane integrity is compromised, LAAO enzymes come into contact with aromatic l‐amino acids found in cryo‐diluents and extenders, leading to deamination and the generation of H_2_O_2_. Consequently, the ROS‐mediated death of some sperm cells can negatively affect and reduce the viability of the remaining live spermatozoa (Gualtieri et al. 2021).

### 3.5 Leukocyte Contamination

Leukocytes in semen contain granulocytes, macrophages and T lymphocytes. Protease, ROS and cytokines released by activated leukocytes can cause LPO and DNA fragmentation (Li et al. 2020). Spermatozoa can be negatively impacted by even trace amounts of leukocytes, especially neutrophils and macrophages, found in semen or extenders. According to Agarwal et al. (2014), these immune cells are triggered when semen is collected, handled, cooled or frozen. In response, they produce a respiratory burst, an enzymatic process that is mediated by NADPH oxidase and produces a significant amount of ROS, including superoxide anion (O_2_•^−^) and its derivatives.

### 3.6 Nitric Oxide (NO) Burst and Reactive Nitrogen Species (RNS)

Elevated concentrations of RNS, particularly peroxynitrite, induce nitrosative stress, a key factor contributing to reduced sperm function (Uribe et al. 2023). NO, a highly reactive gaseous molecule with a short half‐life, is produced through the NADPH‐dependent conversion of l‐arginine to l‐citrulline by nitric oxide synthases (NOSs). However, excessive NO levels have been shown to adversely affect the motility, viability and metabolism of human spermatozoa in vitro (Saeednia et al. 2013). In spite of the fact that this evidence is founded on research in human sperm, the basic processes of nitrosative stress are likely to be preserved among mammalian species. Nevertheless, the degree and level of NO‐toxicity during the cryopreservation process must be directly verified in bovines before drawing direct conclusions.

### 3.7 Oxygen Concentration and Extender Composition

The composition of semen extenders and the concentration of oxygen they contain are the key external factors that influence sperm oxidative stress during cryopreservation. Although oxygen is unquestionably essential for the survival of living cells, it can also give rise to free radicals—highly ROS that interact with key cellular components such as carbohydrates, lipids and proteins. These interactions can damage cellular membranes, ultimately resulting in cell death and aging (Kumar et al. 2019). As oxygen molecules are substrates for both enzymatic and non‐enzymatic redox processes, high concentrations of dissolved oxygen in extenders boost the formation of ROS (Mustapha et al. 2022).

### 3.8 Depletion of Antioxidant Defences

Under regular circumstances, sperm depend on non‐enzymatic antioxidants like reduced glutathione (GSH), vitamins, and thiols as well as enzymatic antioxidants like GPx, CAT and SOD to neutralize ROS and maintain redox homeostasis (Agarwal et al. 2014). However, the intracellular antioxidant pool is significantly reduced throughout the freeze–thaw cycle (Bilodeau et al. 2000). Cryo‐survival rates may therefore be low, with about 50%–60% of spermatozoa maintaining their motility at best.

## 4 ROS‐Associated Decline in Sperm Quality

Excessive ROS can negatively affect sperm function through multiple mechanisms, as they cause damage to critical cellular components, such as lipids, protein and DNA (Wang et al. 2025) (Table 2).

### 4.1 Lipid Peroxidation

LPO in sperm occurs when ROS attack the PUFAs within the sperm plasma membrane, leading to oxidative degradation of lipids. This process disrupts membrane integrity and fluidity, thereby impairing sperm motility, acrosome reaction and the ability to interact with the oocyte (Barati et al. 2020). ROS triggers LPO by reducing intracellular ATP levels, damaging the axoneme and causing structural alterations—effects compromise sperm motility and viability due to the high mitochondrial concentration in the sperm midpiece (Abah et al. 2025). Oxidants, including free radicals and non‐radical species such as superoxide anion, hydrogen peroxide and hydroxyl radicals, target lipids containing carbon–carbon double bonds, particularly PUFAs. This initiates a chain reaction involving hydrogen abstraction and oxygen insertion, producing lipid peroxyl radicals and hydroperoxides. The result is an autocatalytic, self‐propagating process that destabilizes the membrane (Ayala et al. 2014).

When the LPO cascade concludes, highly reactive by‐products such as malondialdehyde (MDA), 4‐hydroxynonenal (4‐HNE), acrolein and isoprostanes are formed. These compounds further compromise cellular integrity by increasing membrane permeability, reducing membrane potential, disrupting mitochondrial function and inducing DNA damage, thereby lowering fertilization potential. Through interactions with DNA bases and the sugar backbone, ROS cause genetic damage characterized by base modifications, abasic sites, chromosomal rearrangements, frameshifts, deletions, DNA–DNA crosslinks and microdeletions (Upadhyay et al. 2022).

### 4.2 Protein Damage

ROS damage structural and functional proteins, oxidative stress is the primary cause of protein alterations in sperm. Being highly dependent on specialized proteins to achieve motility, membrane integrity and fertilizing an egg, spermatozoa are particularly susceptible due to low cytoplasmic antioxidant levels. Under the influence of ROS on some amino acids, such as cysteine, methionine and tyrosine, which contain either sulphur or aromatic groups highly susceptible to oxidation, protein oxidation occurs (Mostek et al. 2018). The oxidative damage of the enzymatic proteins of the energy metabolism, including the glycolytic and mitochondrial enzymes, interferes with the ATP production, required in the motility, capacitation and fertilization. Protein oxidation in sperm targets the cytoskeletal actin and tubulin proteins, which are some of the crucial proteins. The cytoskeleton integrity is disintegrated by the oxidation of actin and tubulin leading to reduced sperm motility and flagellar movement (Wang et al. 2025).

### 4.3 DNA Damage

For successful ART, sperm's DNA integrity is a crucial factor. There's a significant positive correlation between sperm's ROS and DNA damage (Upadhyay et al. 2022). ROS causes damage to sperm DNA which ultimately results in double helix fragmentation of the DNA. Sperm DNA is densely packed and compacted and is wrapped inside protamines. The compaction itself acts as defence mechanism against external damages, but as sperm DNA does not contain enough DNA repair mechanisms available and DNA is loosely packed at some locations, especially those involved in early embryonic development, it remains susceptible to oxidative stress. Base alterations, strand breakage and the development of oxidative lesions are the results of interactions between ROS, especially hydroxyl radicals, and DNA bases (Wang et al. 2025). Hydroxyl radicals extract hydrogen from the deoxyribose sugar by attaching themselves to the double bonds of DNA bases and as a results DNA strands demolish and base releases. The oxidation of guanine produces 8‐OHdG, one of the most well‐known oxidative DNA adducts (Bollwein and Bittner 2018). Damage to the sperm's DNA can result in a number of fertility‐related issues, such as impaired fertilization, impaired embryo development and possibly even spontaneous miscarriage or developmental problems in the offspring (Kamkar et al. 2018). Reduced sperm motility and aberrant morphology are also linked to oxidative DNA fragmentation, which lowers the chance for fertilization (Dorostghoal et al. 2017).

### 4.4 Apoptosis

ROS results in reduced mitochondrial respiration and perturbations of the mitochondrial redox homeostasis, resulting in reduced ATP production and altered calcium homeostasis, ultimately leading to cell death (Avram et al. 2022). It is a crucial mechanism via which cellular damage and oxidative stress lower the quality of sperm and male reproductive capacity.

### 4.5 Mitochondrial Dysfunction

Sperm mitochondria are one of the main ROS generation sites. Dysfunction of mitochondria decreased the activity in sperm, acrosome reaction rate, capacitation ratio and integrity of DNA (Wang et al. 2025). Midpiece sperm mitochondria form a sheath around the axoneme, playing a role on ATP production which is thought to activate and drive sperm motility, so greater numbers of sperm mitochondria are associated with faster swimming (Costa et al. 2023).

### 4.6 Premature Capacitation and Acrosome Reaction

The loss of ABHD16B during sperm cryopreservation disrupts membrane lipid balance (DAG and PC), making sperm more susceptible to ROS‐induced damage and premature capacitation and acrosome reaction, and therefore, infertility in males. The physiological and biochemical changes in spermatozoa required to attain fertilization competency are collectively referred to as ‘capacitation’ (Takei 2024). Spontaneous acrosome reaction (sAR) can occur in the sperm before binding to the oocyte cumulus oophorus and, therefore, reduce the ability to fertilize (Breitbart and Grinshtein 2023).

### 4.7 Impairment of Sperm‐Oocyte Interaction

Overproduction of ROS may impair sperm‐oocyte interaction by promoting LPO, oxidation of proteins and structural destruction of a sperm plasma membrane, such as acrosomal region, which is critical in zona pellucida penetration. Such oxidative changes disrupt sperm motility, capacitation and acrosome reaction and eventually diminish the potential to achieve fertilization (Wang et al. 2025; Kaltsas 2023).

## 5 Mitigation Strategies of ROS

Mitigation of ROS in sperm focuses on protecting cells from oxidative damage and preserving their fertilizing potential. This can be achieved through supplementation with antioxidants, plant extract, nanoparticles, mitochondria‐targeted antioxidants and non‐antioxidant approaches ensuring better motility, membrane integrity and overall sperm quality. Interestingly, the various techniques differ greatly in how they operate, their effectiveness and practicality. Enzymatic antioxidants, such as SOD and CAT, act by direct removal of primary ROS, but as they are proteinaceous in nature, they do not have the capability to permeabilize membranes. However, non‐enzymatic antioxidants are able to penetrate membranes readily, yet their antioxidant capacity and optimum dosing vary greatly. Plant extracts have a multi‐target protective effect that is cost‐effective and contains compounds, including polyphenols and flavonoids, but the non‐uniformity of the phytochemical profiles is an issue in standardization. The nanoparticles have the potential to improve targeted delivery and enable neutralization of ROS with time, although there is a concern of cytotoxic effects when at a high concentration. In the meantime, mitochondria‐directed antioxidants provide a more focused approach with a more direct target of the primary intracellular source of ROS, but more optimizing of dosage and formulation is required before broad use. Thus, a comparative analysis of these methods is extremely important to develop rational and evidence‐based supplementation strategies.

### 5.1 Enzymatic Antioxidants

Enzymatic antioxidants, such as SOD, CAT and GPx, are central to reducing ROS‐induced damage in cryopreserved bovine sperm. A single freeze–thaw cycle markedly decreases endogenous defences (↓SOD, ↓GSH), leading to LPO and loss of function. The addition of SOD in diluter reduces the production of MDA and showed significantly enhanced sperm vitality (Asadpour et al. 2012). CAT detoxifies H_2_O_2_ generated during cooling; addition of CAT has been shown to enhance motility, acrosome integrity and reduce MDA levels (Eidan 2016). The GPx system, particularly GPx4, protects membranes by reducing lipid hydroperoxides, and its activity correlates positively with sperm motility and cryo‐survival (Asadpour et al. 2012). Beyond these, peroxiredoxins (PRDXs) act as supplementary peroxide scavengers, with PRDX5/6 translocation during freezing indicating their role in sperm oxidative defence (Agnieszka et al. 2025). Collectively, optimizing enzymatic antioxidant activity in extenders lowers ROS load, stabilizes membranes and mitochondria and improves fertility outcomes (Table 1). A stringent assessment of the evidence at hand points to a number of limitations. The majority of the benefits of exogenous enzyme antioxidants in bovine semen have been reported on single‐laboratory studies with small sample sizes and have yet to be independently validated. Moreover, the preferred dosing range in the literature is diverse not only between different studies but also among bull breeds, meaning that the variability within individuals, which is already a known problem in bovine semen cryopreservation, could have a potent impact on enzyme supplementation reactions. Exogenous proteins, such as SOD and CAT, are also poorly membrane‐permeable and inherently limited and thus act primarily in the extracellular space rendering them less effective in intracellular or mitochondrial ROS. Combined, these limitations highlight the necessity of multi‐breed dose‐optimization studies that are conducted in a standardized manner and the creation of formulations that enhance the membrane permeability of enzymatic antioxidants.

**TABLE 1:** Effect of enzymatic antioxidants in reducing reactive oxygen species (ROS)‐induced damage in cryopreserved bovine sperm.

Enzymatic antioxidant	Mode of action	Dose	Observed effects	References
Superoxide dismutase (SOD)	Converts O_2_•^−^ into H_2_O_2_ and O_2_	0.1 mg/mL 100 U/mL	Enhances post‐thaw motility and viability The addition of SOD results in the lowest MDA levels	Sapanidou et al. (2023) Asadpour et al. (2012)
Catalase (CAT)	Decomposes H_2_O_2_ into H_2_O and O_2_	100 IU/mL	CAT supplementation improves sperm quality (motility, viability, plasma membrane integrity, and acrosome integrity) but increases MDA levels	Eidan (2016)
Glutathione peroxidase (GPx)	Reduces H_2_O_2_ and lipid hydroperoxides to water and corresponding alcohols	50 U/mL	GPx produces the highest sperm viability and the lowest MDA levels	Asadpour et al. (2012)

Abbreviation: MDA, malondialdehyde.

### 5.2 Non‐Enzymatic Antioxidants

Non‐enzymatic antioxidants play a crucial role in protecting bovine sperm from ROS‐induced cryodamage. Various researchers have utilized different types of non‐enzymatic antioxidants (Table 2). Vitamin E (α‐tocopherol), a lipid‐soluble antioxidant, and its supplementation in extenders improve motility and viability and reduce the MDA and H_2_O_2_ production (Motemani et al. 2017).

**TABLE 2:** Effect of non‐enzymatic antioxidants in reducing reactive oxygen species (ROS)‐induced damage in cryopreserved bovine sperm.

Non‐enzymatic antioxidant	Mode of action	Dose	Observed effects	References
Vitamin E (α‐tocopherol)	Scavenges lipid peroxyl radicals	4.8 mM	Higher motility, viability and membrane functionality Lower MDA and H_2_O_2_ production	Motemani et al. (2017)
Vitamin C (ascorbic acid)	Scavenges ROS	5 mM 2.5 mmol/mL	Live spermatozoa, acrosomal integrity and HOST‐positive cells increase, whereas sperm abnormalities and MDA levels decrease; SOD levels increase Higher motility, plasma and acrosomal membrane integrity	Singh et al. (2020) Pinto et al. (2020)
Glutathione (GSH)	Detoxifies peroxides and maintains redox balance in sperm	2.5 mmol/L 2 mM/mL 2.5 mmol/mL	GSH enhances mitochondrial membrane potential and viability while reducing DNA fragmentation Higher motility, acrosomal, plasma membrane integrity and mitochondrial potential and lower oxidative stress percentage Improves motility, mitochondrial activity, acrosomal integrity	Hu et al. (2016) Almeida et al. (2021) Pinto et al. (2020)
l‐Carnitine	Enhances mitochondrial fatty acid metabolism	0.05 mg/mL	Motility, HOST‐reactive sperm and viability increase, whereas MDA, SOD and alanine aminotransferase levels do not differ significantly	Vaghasiya et al. (2024)
Taurine/Hypotaurine	Scavenge ROS and protect plasma membrane from lipid peroxidation	50 mM	Sperm viability, acrosomal and plasma membrane integrity, progressive motility and cholesterol content increase, whereas morphological and nuclear abnormalities and enzyme leakage decrease	Ponraj et al. (2022)
Melatonin	Potent free radical scavenger	100 µM	Improves motility, viability and antioxidant status	Lavrentiadou et al. (2023)
Coenzyme Q10	Regenerates vitamin E and supports ATP production	30 µM	Increases motility, viability, plasma membrane integrity Decrease sperm abnormalities	Saeed et al. (2016)
Crocin	Scavenges superoxide anion Induces TAC	1 mM	Show higher CASA kinematic values and positive effect on motility Inhibition of LPO	Sapanidou et al. (2022)
Crocetin	Quenches ROS and enhances the intracellular antioxidant capacity	2.5 μМ	Reduces the production of superoxide anion, hydrogen peroxide and lipid peroxidation Improves motility, viability and acrosomal integrity	Sapanidou et al. (2016)
Quercetin	Potent free radical scavenger Inhibits lipid peroxidation	25 µg/mL	Positive effect on sperm DNA integrity does not improve progressive and total motility, plasma membrane integrity or sperm abnormalities	Avdatek et al. (2018)
Resveratrol	Reduces mitochondrial ROS	0.05 mM	Greater sperm progressive motility, average path velocity, straight linear velocity, linearity and straightness	Assunção et al. (2021)
Curcumin	Scavenges superoxide and hydroxyl radicals	25 or 50 µM	Elevates GSH and mitochondrial membrane potential while reducing ROS and malondialdehyde concentration	Lin et al. (2025)
Lycopene	Quenches singlet oxygen	1.5 mmol/L	Improves membrane and acrosomal integrity Decreases production of ROS, protein carbonyl and lipid peroxidation	Tvrda et al. (2017)
Catechin	Stabilizes membranes	25 µg/mL	Protects morphological and DNA integrity from cryodamage and increases total antioxidant activity (TAC)	İnanç et al. (2019)
Epigallocatechin gallate	Inhibits ROS production	20 µmol	Increases in TAC and decreases in MDA	Parvizi et al. (2024)
Epicatechins	Scavenges superoxide and hydroxyl radicals Decreases LPO	1–50 μМ/L	Preserves motility and viability Reduces ROS production	Greifová et al. (2017)
Genistein	Inhibit the oxidative stress	60 µg/mL	Improves progressive motility Preserves plasma and acrosome membrane integrity Reduces MDA production	Liang et al. (2025)
Lactoferrin	Chelate‐free iron	100 µg/mL	Improves viability and motility	Kobayashi et al. (2021)

Abbreviation: SOD, superoxide dismutase.

Similarly, inclusion of 5 mM vitamin C with extender significantly increased live spermatozoa, acrosomal integrity and HOST positive spermatozoa, while significantly decreasing sperm abnormalities and MDA levels (Singh et al. 2020). Glutathione is the primary intracellular non‐protein thiol molecule that scavenges ROS directly, and its incorporation reduced ROS levels and DNA fragmentation, preventing premature capacitation (Almeida et al. 2021). l‐Carnitine is an important molecule that plays multiple roles in intermediate metabolism. The high concentration of carnitine detected in the male reproductive system, notably in the epididymis, suggests its critical function in energy metabolism and sperm maturation Vaghasiya et al. (2024) showed that at the post‐thawed stage, motility, HOST‐reactive sperm and viability were significantly higher in the l‐carnitine groups, whereas sperm abnormalities were lower compared to the control. Taurine, a sulfonic amino acid and non‐enzymatic scavenger, protects spermatozoa from LPO. The extender containing taurine improved viability, acrosomal integrity, plasma membrane integrity, motility, sperm cholesterol content and sperm kinetics and velocity (Ponraj et al. 2022). Melatonin is an amphiphilic hormone, acts as a potent antioxidant that stabilizes mitochondrial function and protects sperm DNA, thereby improving motility and reducing apoptosis during cryopreservation (Ofosu et al. 2021). With the incorporation of melatonin the percentage of motile and fast spermatozoa is increased (Lavrentiadou et al. 2023). Coenzyme Q10 is mostly found in the mitochondria of sperm cells, and its levels in the seminal fluid correlate with sperm properties. Integration of CoQ10 in Tris‐diluter improves sperm mobility, liveability, plasma membrane integrity and reduces abnormalities. It also protects against acrosomal damage in cattle spermatozoa (Saeed et al. 2016). Crocin and crocetin, bioactive carotenoids from saffron, are potent antioxidants that scavenge ROS, protect sperm DNA and stabilize membranes. The application of crocin and crocetin effectively preserved sperm quality during the entire period of in vitro incubation (Sapanidou et al. 2016, 2022). Quercetin is a flavonoid antioxidant improves sperm DNA integrity and reduces GSH depletion and increased SOD and GPx activity (Avdatek et al. 2018). Resveratrol, a polyphenol present in numerous types of fruits, has powerful antioxidant capabilities due to its radical scavenger activity, resulting in a reduction in ROS formation and LPO. Pre‐treatment of spermatozoa with resveratrol reduced ROS and MDA levels, maintaining viability, acrosomal integrity and mitochondrial activity (Assunção et al. 2021). Curcumin is a plant‐based polyphenol, mostly derived from turmeric rhizomes. Natural curcumin has eight times the antioxidant activity of vitamin E and 2.75 times that of vitamin C. Curcumin can transfer electrons or give hydrogen atoms, scavenging reactive free radicals, triggering cytoprotective signals and reducing oxidative damage to cells. Curcumin regulates antioxidant activity by scavenging hydrogen peroxide and its dosages in diluter increased GSH and MMP levels and decreased ROS and MDA levels and improved fertilization (Lin et al. 2025). Lycopene supplementation significantly improved sperm motility and preserved mitochondrial activity, reduced ROS and superoxide generation and decreased protein carbonyls, LPO and oxidative DNA damage compared to control (Tvrda et al. 2017).

Catechin (CT) is an antioxidant polyphenol found in plants: epigallocatechin 3‐gallate (EGCG), epicatechin and epigallocatechin are examples of important dietary CTs. Their inclusion in semen extender protects cells from the damage produced by unstable chemicals known as free radicals and improved sperm motility, viability and reduces ROS generation, protein carbonyls, LPO and oxidative DNA damage (İnanç et al. 2019; Parvizi et al. 2024; Greifová et al. 2017). Supplementing the semen extender with genistein could improve the antioxidant performance of sperm in bovine semen, inhibit oxidative stress during cryopreservation, alleviate freeze–thaw damage of sperm acrosomes and mitochondria, and improve the motility performance of sperm after thawing (Liang et al. 2025). Lactoferrin is secreted from the epididymis, prostate gland, and seminal gland (Belleannée et al. 2011). Lactoferrin acts mainly by chelating free iron, thereby preventing ROS generation through the Fenton reaction. It also stabilizes cell membranes and protects sperm from oxidative stress–induced damage. Lactoferrin reduced early declines in sperm motility and promoted capacitation‐related changes. Under oxidative stress, it protected against loss of flagellar and progressive movement. In frozen–thawed sperm, it improved survival and reduced agglutination (Kobayashi et al. 2021). In general, the scientific literature on the use of non‐enzymatic antioxidants in bovine sperm cryopreservation is large but very inconsistent. Although a broad range of compounds and dosages have been reported to have beneficial effects, direct comparative studies conducted in the same experimental system are scarce, and it is difficult to establish an unambiguous order of efficacy. Moreover, other studies show positive effects at specific concentrations only and the inhibitory or even cytotoxic effects at high concentrations, which also emphasize the paramount significance of dose optimization. Lipid‐soluble antioxidants (vitamin E, lycopene and curcumin) seem to have a constant negative effect on membrane LPO, compared with more variable effects of the water‐soluble antioxidants (vitamin C and glutathione) which might be due to variations in the extender composition and dilution procedures. It would be a major boost to the evidence to do further comparative studies in the future using consistent assessment parameters—such as sperm motility, DNA fragmentation index and MDA levels—in a sample of bulls.

### 5.3 Plant Extract

Medicinal plants are gaining popularity in andrology due to their significant health benefits (Table 3). Proper extraction and assessment of plants and herbs is vital for exploring their antioxidant potential. Plant extracts are a source of natural antioxidants, and they have gained attention as a cheap and natural source of additives for protecting and improving sperm functions during semen cryopreservation (Tavares et al. 2021). Many plant extracts contain variety of polyphenols, flavonoids and bioactive compounds, which act as antioxidants and neutralize the ROS, decrease LPO and stabilize sperm membranes, thereby enhancing the quality of post‐thaw semen. A wide range of plants and herbs have been investigated for their protective potential in semen cryopreservation. For example, *Rosmarinus officinalis, Zingiber officinale, Calendula officinalis, Vitis vinifera, Sambucus nigra, Schisandra chinensis, Moringa oleifera, Albizia harveyi, Diospyros kaki, Eurycoma longifolia, Origanum vulgare, Urtica Dioica, Punica granatum, Syzygium cumini, Pinus Brutia* Ten. and *Levisticum officinale* are reported to have higher antioxidant activity and reduced the oxidative stress and increased the total sperm motility, progressive sperm motility, sperm viability and sperm plasma membrane integrity and decreased ROS production, protein oxidation and LPO in post‐thaw bull semen (Yeni et al. 2018; Merati and Farshad 2020; Benko et al. 2019; Sapanidou et al. 2014; Abramanov et al. 2017; Tvrdá et al. 2020; Authaida et al. 2025; Sobeh et al. 2017; El Sheshtawy and El‐Nattat 2017; Baiee et al. 2018; Daghigh Kia et al. 2016; Mohamed and Abdulkareem 2020; El‐Sheshtawy et al. 2016; Suleman et al. 2020; Taşdemir et al. 2020; Tvrdá et al. 2019). The findings of different independent studies conducted using various plant species are congruent in showing that polyphenol‐ and flavonoid‐rich extracts possess a strong cryoprotective activity, which is mainly achieved through the action of an antioxidant and membrane stabilizers. Nevertheless, despite the general enhancement in post‐thaw sperm quality of plant extracts, the effective concentrations can be diverse, depending on the nature of the plant source, the extraction procedure (aqueous, ethanolic or methanolic), and the type of solvent, so direct comparisons are difficult.

**TABLE 3:** Effect of plant extract in reducing reactive oxygen species (ROS)‐induced damage in cryopreserved bovine sperm.

Plant extract	Mode of action	Dose	Observed effects	References
*Rosmarinus officinalis*	Reduces lipid peroxidation	5 or 10 g/L 25 or 50 µg/mL	Positive effects on total motility, average path velocity, viability and HOST Increases GPx activity Does not enhance sperm progressive and total motility Reduces MDA levels and chromatin damage	Daghigh‐Kia et al. (2014) Yeni et al. (2018)
*Zingiber officinale*	Stabilizes membranes	10 or 20 mg/L	Improves motility, velocity parameters, acrosomal integrity, mitochondrial activity Decreases LPO	Merati and Farshad (2020)
*Calendula officinalis*	Scavenges superoxide radicals	75 or 150 µg/mL	Increases motility and mitochondrial activity Decreases ROS levels, protein oxidation and membrane damage caused by LPO	Benko et al. (2019)
*Vitis vinifera*	Free radical scavenger	5 µg/mL	Maintains motility, viability and acrosomal Integrity Lower MDA level	Sapanidou et al. (2014)
*Sambucus nigra*	High antioxidant activity at low concentration	5 or 10 µg/mL	Promotes sperm motility and mitochondrial Activity Reduces ROS production	Abramanov et al. (2017)
*Schisandra chinensis*	Antioxidant activity	5–50 µg/mL	Improves motility, mitochondrial activity and TAC Decreases ROS production	Tvrdá et al. (2020)
*Moringa oleifera*	Scavenges ROS Reduces lipid peroxidation	1 mg/mL	Improves motility, viability and membrane integrity	Authaida et al. (2025)
*Albizia harveyi*	Reduce oxidative stress	1.5 µg/mL	Improves sperm motility, viability, membrane integrity and TAC Reduces abnormality, chromatin damage and MDA	Sobeh et al. (2017)
*Diospyros kaki*	Scavenging ROS and lipid peroxidation	2% or 4%	Improves sperm motility, viability and membrane integrity Higher conception rate	El‐Sheshtawy and El‐Nattat (2017)
*Eurycoma longifolia*	Reduces lipid peroxidation	5 mg/mL	Improves membrane integrity, motility and viability Reduces DNA damage	Baiee et al. (2018)
*Origanum vulgare*	Reduce in lipid peroxidation	2 or 4 mL/dL extender	Improves the motility, viability and plasma membrane integrity Increases the activity of SOD and CAT	Daghigh Kia et al. (2016)
*Urtica dioica*	Membrane stabilizer	0.01 g or 0.02 g/50 mL	Enhances sperm motility, viability, plasma membrane and acrosome integrity Decreases abnormality	Mohamed and Abdulkareem (2020)
*Punica granatum*	Scavenges ROS	10% or 20%	Increases motility and viability	El‐Sheshtawy et al. (2016)
*Syzygium cumini*	Antioxidant protection via polyphenols and flavonoids	7 or 14 ppm	Improves sperm membrane integrity, motility and fertilizability	Suleman et al. (2020)
*Pinus brutia* Ten.	Scavenges ROS	50 µg/mL	Shows the lowest malondialdehyde levels and the highest glutathione levels Does not have a positive effect on sperm motility	Taşdemir et al. (2020)
*Levisticum officinale*	Scavenges ROS	37.5 µg/mL	Improves motility and antioxidant capacity	Tvrdá et al. (2019)

Abbreviations: GPx, glutathione peroxidase; MDA, malondialdehyde; SOD, superoxide dismutase; TAC, total antioxidant activity.

The majority of the studies are carried out in controlled laboratory conditions with few bulls and this may not be reflective of the biological variation in the field. Further, there is an abundance of evidence on short‐term in vitro findings with limited in vivo fertility trials to validate real changes in conception rates. The exact bioactive compounds that cause these effects are not well established, and the fact that extraction methods, geographical location and harvesting are different, impedes standardization. Moreover, possible cytotoxicity with increased dosage underscores the need to optimize on a case‐by‐case basis before it can be practically utilized.

### 5.4 Nanoparticles

**FIGURE 2:**
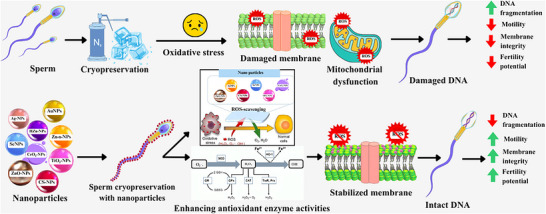
Nanoparticles bind to ROS components in cryopreserved sperm. The picture illustrates two nanoparticle action mechanisms in freeze bovine sperm. Category I (inert nanoparticles, e.g., chitosan and lipid nanoparticles) coats and delivers traditional antioxidant molecules (vitamin E and glutathione) to the surface of the sperm to increase bioavailability and decrease oxidative damage to the membrane, but uncatalytically. CeO_2_‐NPs are Category II nanozymes and are similar to the enzymatic antioxidant activity, decomposing superoxide and hydrogen peroxide via surface redox cycling. The two categories have the same downstream effects, that is, reduced lipid peroxidation, maintained mitochondrial membrane potential, lessened DNA fragmentation and enhanced post‐thaw motility. It is important to note that the efficacy and safety of nanoparticles are associated with the size of the particle, surface chemistry and dose administered, and adverse effects have been reported beyond the optimal range. GPx, glutathione peroxidase; ROS, oxygen species.

Antioxidants based on nanotechnology are emerging as one of the best bovine semen cryopreservation agents because of their high delivery capability and high ROS‐scavenging capacity (Figure 2). These nanomaterials can be further divided into two functionally distinct groups based on their mode of action, that is, carrier‐based/indirectly acting nanomaterials, and intrinsically bioactive nanomaterials (nanozymes) (Saadeldin et al. 2020). Nanomaterials that fall within the first category are chitosan nanoparticles (CS‐NPs) and humic–zinc nanoparticles (HZn‐NPs), which are mostly utilized as delivery carriers or structural stabilizers (Mikusova and Mikus 2021). These compounds do not cause antioxidant reactions but increase sperm preservation by allowing bioactive chemicals to be delivered to specific locations, stabilizing plasma membranes or indirectly increasing the activity of natural antioxidant enzymes, such as GPx and SOD (Mahmoud et al. 2023; Alfattah et al. 2025). Their protective effects are therefore more reliant on what bioactive cargo they carry or which cellular pathways they modulate and not on some inherent enzyme property (Saadeldin et al. 2020). The second category consists of nanozymes that have intrinsic enzyme‐like activities; they have the ability to regulate oxidative stress directly without exogenous antioxidants. They can be either prooxidant or antioxidant catalysts. Simulation of GPx with selenoenzymes, including GPx and thioredoxin reductase (TrxR) is simulated by selenium nanoparticles (SeNPs) (Kondaparthi et al. 2019), and simulation of a variety of enzymes, including SOD, CAT, and peroxidase, is simulated by cerium oxide nanoparticles (CeO_2_‐NPs) through Ce3+/Ce4+ cycling (Xu and Qu 2014). Likewise, ZnO‐ and Zn‐based nanoparticles assist in antioxidant defence, CAT‐enhancing activity and raising endogenous SOD (Wang et al. 2024). TiO_2_‐NPs, gold nanoparticles (AuNPs) and AgNPs are also endowed with inherent nanozyme activity but TiO_2_‐NPs and in some circumstances, AuNPs and AgNPs can also support the formation of ROS as a result of their pro‐oxidant or context‐dependent catalytic actions. This can have a detrimental effect on sperm functionality (Tandon et al. 2020; Taylor et al. 2014; Pande et al. 2022). Existing data indicate that nanomaterials have two context‐specific effects (Zhu et al. 2025). Some nanomaterials are able to replicate antioxidant enzymes, such as SOD and CAT, or induce endogenous defences, such as GPx, which reduces the levels of ROS and enhances sperm motility, membrane integrity and DNA stability. On the other hand, some nanomaterials—including CeO_2_, ZnO, MnO_2_, Mn_3_O_4_, SiO_2_, iron oxide (IO), carbon (C), gold (Au), silver (Ag) and curcumin (Cur)—have been found to cause reproductive and genotoxic toxicity even with relatively low doses or after short‐term exposure in humans and mice (Pande et al. 2022; Taylor et al. 2014; Zhu et al. 2025). The degree of these adverse effects will depend on a number of physicochemical parameters, like material composition, particle size, surface properties, dosage and exposure time. The functionalization of surfaces, such as antioxidant loading and polymer coating, can contribute greatly to the regulation of these consequences. Notably, much of the toxicity data is in the literature of environment exposures or high dosage situations, which are not directly relevant to the controlled, low dose exposure of nanoparticles in sperm cryopreservation studies. Li et al. (2023), Kanwar et al. (2023), Ram et al. (2025), Khalil et al. (2019), Alfattah et al. (2025) and Isaac et al. (2017) showed that nanoparticles, used at biologically relevant levels, did not cause any apparent toxicity to bull sperm. Nevertheless, the encouraging results obtained with nanoparticles in the cryopreservation of bovine sperm must be taken cautiously. Strict dosage optimization, application of surface modification measures to reduce cytotoxic effects and extensive assessment of genotoxicity are essential conditions prior to the safe application of these materials in commercial breeding programmes (Table 4).

**TABLE 4:** Effect of nanoparticles in reducing reactive oxygen species (ROS)‐induced damage in cryopreserved bovine sperm.

Name of nanoparticles	Mode of action	Dose	Consequence on sperm	References
Selenium nanoparticles (SeNPs)	Enhance glutathione peroxidase activity	1 µg/mL 1 or 2, or 4 µg/mL	Increases motility, livability, plasma membrane integrity and chromatin integrity Reduce abnormality, apoptosis, LPO and sperm damage Increase motility, viability, mitochondrial activity, plasma membrane integrity and acrosome integrity Enhance antioxidant capacity and embryonic development potential	Khalil et al. (2019) Li et al. (2023)
Zinc oxide nanoparticles (ZnO‐NPs)	Stabilize plasma membrane	1 µg/mL	Increase motility, viability, plasma membrane and acrosome integrity Higher TAC Reduce abnormality and MDA	Ram et al. (2025)
Zinc nanoparticles (ZN‐n‐NPs)	Enhance antioxidant defence	2.97 mol/mL 2.7 mol/mL	Improve plasma membrane integrity preserve mitochondrial activity Do not affect progressive motility, sperm viability, DNA fragmentation or pregnancy rate	Jahanbin et al. (2021)
Cerium oxide nanoparticles (CeO_2_‐NPs)	Mimic catalase/SOD activity	75 µg/mL	Enhance motility and viability Reduces abnormality	Al‐Janabi et al. (2024)
Titanium dioxide nanoparticles (TiO_2_‐NPs)	Stabilize membranes	10–20 µg/mL	Reduce motility, viability and plasma membrane integrity	Pande et al. (2022)
Gold nanoparticles (AuNPs)	Antioxidant and anti‐apoptotic	10 µg/mL	Reduce motility	Taylor et al. (2014)
Chitosan nanoparticles (CS‐NPs)	Scavenge free radicals	20 µg/mL	Enhance sperm capacitation and acrosome reaction	Mahmoud et al. (2023)
Humic–zinc nanoparticles (HZn‐NPs)	Prevent DNA damage	20 µg/mL	Improve motility, viability, acrosome integrity and various kinematic parameters Increase TAC and SOD and reduce MDA, NF‐jB, H_2_O_2_ and NO Decrease apoptosis and necrosis	Alfattah et al. (2025)
Silver nanoparticles (AgNPs)	Stabilize plasma membrane	125 µg/mL	Increase motility Reduce LPO and ROS	Kanwar et al. (2023)

Abbreviations: LPO, lipid peroxidation; MDA, malondialdehyde; SOD, superoxide dismutase; TAC, total antioxidant activity.

### 5.5 Mitochondria‐Targeted Antioxidants

Mitochondria are the primary source of ROS in sperm, making mitochondria‐targeted antioxidants a promising approach to improve cryopreservation outcomes. Mitoquinone (MitoQ), a coenzyme Q10 derivative conjugated to triphenylphosphonium, selectively accumulates in mitochondria and reduces oxidative stress, thereby improving motility, membrane integrity and mitochondrial activity in cryopreserved bull sperm. One research investigated the impact of varying concentrations of MitoQ (5, 50, 500 and 1000 nM) added to the semen extender on the post‐thaw quality of Holstein bull sperm. The findings revealed that MitoQ supplementation, particularly at 50 and 500 nM concentrations, significantly improved sperm motility, membrane integrity, mitochondrial activity and viability. Additionally, MitoQ effectively reduced MDA levels and ROS concentrations, indicating its potential to alleviate oxidative stress during cryopreservation (Nazari et al. 2025). This finding suggests that targeting antioxidants directly to mitochondria provides a more efficient strategy for preventing ROS accumulation, preserving bioenergetics, and improving post‐thaw sperm functionality. In comparison of mitochondria‐targeted antioxidant and other antioxidant techniques, critical comparative analysis is very important. Conventional antioxidants such as vitamin E, vitamin C and glutathione neutralize ROS in the extracellular or cytoplasmic environment, not specifically targeting mitochondria, which is the primary location of ROS generation in sperm cells. MitoQ and MitoTEMPO, on the other hand, are mitochondria‐targeted compounds that can be accumulated in the mitochondrial matrix several hundred‐fold using the mitochondrial membrane potential (which is about −180 mV). Such a specific subcellular localization, and not variations in pharmacokinetics or intrinsic antioxidant strength alone, is their main mechanistic advantage. Consequently, they are more efficient in inhibiting mitochondrial superoxide generation, maintenance of mitochondrial membrane potential, maintenance of sperm bioenergetics as compared to traditional antioxidants with the same molar concentration.

### 5.6 Non‐Antioxidant Approaches

Although antioxidant methods predominantly counteract the effects of ROS, non‐antioxidant methods aim at decreasing the production of ROS at the origin of the cellular stress, instead of the direct elimination of those free radicals, during sperm cryopreservation. Nonetheless, in recent studies more attention has been given to more sophisticated methods like nanoparticles and plant‐based antioxidants. Thus, only the most important non‐antioxidant techniques have been briefly addressed in this section. Energy substrates, including glucose, fructose and pyruvate, are some of the oldest and most frequently used non‐antioxidant strategies that have been incorporated into bull semen extenders to address metabolic requirements of sperm when equilibrating and in post‐thawing conditions (Djemouai Boukhalfa et al. 2024). The most common membrane stabilizers in bull extenders are egg yolk and soy (soybean) lecithin which have been linked with enhanced post‐thaw motility and membrane integrity in comparative studies of animal based and non‐animal‐based extenders (Venkatesh et al. 2024). It is important to note that recent findings show that plasma of egg yolk (EYP) instead of whole egg yolk improves sperm protection because EYP offers superior membrane stabilization and minimizes the ability of cryo‐induced ROS formation and removal of some lipoproteins or granules that could adversely impact sperm activity (Corcini et al. 2016). Pre‐treating soybean lecithin extenders with cholesterol‐loaded cyclodextrin (CLC) has been shown to enhance post‐thaw motility, membrane activity and acrosome integrity, which are in line with cold shock resistance. It also raises the quantity of cholesterol in the sperm membranes (Venkatesh et al. 2024).

The maintenance of oxygen tension during sperm manipulation, especially under low oxygen states, can reduce the excess production of ROS as a direct consequence of oxygen availability is the oxidative activity ROS generally produces via the oxygen‐dependent metabolic activities, especially by mitochondrial activity (Fleming and Thomson 2025). Reduced dissolved oxygen levels in semen diluter minimize oxidative stress by reducing the generation of ROS and thus improve sperm quality during cryopreservation (Kumar et al. 2018).

In addition to oxygen control, cellular composition of the semen sample per se is also a major determinant of ROS burden. Sperms that are morphologically abnormal and immature especially with excessive cytoplasmic residue are primary source of ROS (Takeshima et al. 2018). Removal of defective or dead spermatozoa through selection techniques, such as swim‐up or density gradient centrifugation, also contributes to lowering overall ROS levels Semen sorting with swim‐up, density‐gradient centrifugation and microfluidic technique is useful to select healthy sperm with higher motility, higher DNA integrity and as well with less amount of harmful ROS molecules production (Wen et al. 2025).

Adequate control of seminal plasma, such as partial removal or controlled dilution of seminal plasma, lowers the occurrence of pro‐oxidant factors. The antioxidant defences of seminal plasma and extracellular vesicles regulate the ROS balance of sperm (Sengupta et al. 2026). Nevertheless, pre‐treatment with seminal plasma prior to the refrigeration procedure enhances sperm quality by lowering post‐thawing ROS production, and fertilization in caprine cryopreserved semen (Esteve et al. 2025).

Another significant yet relatively ignored cause of ROS generation during bull sperm cryopreservation is osmotic stress. The introduction and elimination of penetrating cryoprotectants, in particular, glycerol, leads to fast osmotic changes in spermatozoa, which damages the membrane stability and provokes the formation of free radicals (Guthrie et al. 2002). The effects of the cryoprotectants are normally reduced by a gradual equilibration of the sperm cells with the penetrating sperm cells like glycerol or DMSO. The gradual introduction enables the spermatozoa to gradually get used to the change in osmotic and prevents the massive changes in volume. Nevertheless, high doses or fast exposure to cryoprotectants can be toxic and destabilizing to membranes. Glycerol can cause pathological changes to sperm membrane lipid structures by toxic, chemical and osmotic mechanism (Otero et al. 2025).

To solve these cumulative sources of cryodamage it is finally necessary to have an accurate control over the actual freezing procedure. In the normal process of semen freezing, the rate of chilling of spermatozoa is not regulated, which exposes them to a higher risk of intracellular ice crystals, osmotic stress and oxidation damages. To address these restrictions, more sophisticated freezing methods such as programmable freezers and vitrification processes have been designed to create a more accurate control of the temperature reduction in the cryopreservation process. Neubert et al. (2025) investigated the effect of freezer and straw positioning on the post‐thaw quality of bull spermatozoa and discovered that freezer type has a significant impact on post‐thaw bull sperm quality, with TurboFreezer M achieving the best values for total motility and acrosome‐intact spermatozo, but straw position (top, centre and bottom) had no significant quality effects. The vitrification technology has also become popular because of its high cooling rate and minimized amount of physical damage to sperm cells. Alternative to the conventional slow freezing methods, vitrification can be used because it removes the damage caused by ice crystals in cells (Amini and Benson 2023).

## 6 Conclusion

Cryopreservation‐induced oxidative stress remains one of the major challenges in maintaining bovine sperm quality and fertility. ROS, generated from both endogenous metabolic activity and cryopreservation‐related stressors, cause LPO, protein oxidation, DNA fragmentation and mitochondrial dysfunction, ultimately compromising sperm function. Extensive research over the past decades has demonstrated that strategic incorporation of enzymatic and non‐enzymatic antioxidants, plant‐derived extracts, nanoparticles, non‐antioxidant approaches and mitochondria‐targeted molecules can effectively mitigate ROS‐induced damage. Together, these multifaceted strategies not only improve post‐thaw motility, viability and fertilizing potential but also pave the way for more efficient and reliable bovine reproductive technologies. In my perspective, MitoQ and MitoTEMPO are the most promising since they target mitochondria, which is critical. Nanoparticle‐based systems and extracts of plants are emerging as promising alternatives, but their efficacy, in most cases, is dependent on the species and dosage, and the cytotoxicity of the nanoparticles at high doses has yet to be determined, and thus, their application can be expanded. In spite of these developments, there are still some key gaps. The optimal antioxidant ratios and dosage levels in different breeds and individual bulls are not well known, and most of the existing studies are done under controlled laboratory conditions that may not translate to the conditions of commercial cryopreservation. Additionally, the majority of the mitigation mechanisms have been investigated independently; future studies should strive to integrate them in a synergistic approach so as to target two or more of the ROS pathways simultaneously. It would also lead to a greater standardization of outcome measures across studies, which would promote the comparability. The bovine‐specific research that will be developed to overcome these limitations will be of great significance as it will be required to bring the available knowledge into practical solutions to improve the artificial insemination and in vitro fertilization programmes that are used in the cattle industry.

## Author Contributions

**Mst. Mahomudha Akhtar**: conceptualization and design, literature search and data collection, drafting and writing the manuscript, prepared the figures and tables, supervision and guidance. **Sakib Hossain**: literature search and data collection, manuscript writing, prepared the figures. **Afia Mahmuda Meem**: literature search and data collection, manuscript writing. **Afiya Fairuz Lubaba**: literature search and data collection, manuscript writing. **Md. Kamrul Hasan Kazal**: conceptualization and design, literature search and data collection and writing the manuscript, supervision and guidance.

## Funding

The authors have nothing to report.

## Conflicts of Interest

The authors declare no conflicts of interest.

## Data Availability

Data sharing not applicable to this article as no datasets were generated or analysed during the current study.
